# A Non Intrusive Human Presence Detection Methodology Based on Channel State Information of Wi-Fi Networks

**DOI:** 10.3390/s23010500

**Published:** 2023-01-02

**Authors:** Carlos M. Mesa-Cantillo, David Sánchez-Rodríguez, Itziar Alonso-González, Miguel A. Quintana-Suárez, Carlos Ley-Bosch, Jesús B. Alonso-Hernández

**Affiliations:** 1 Institute for Technological Development and Innovation in Communications, University of Las Palmas de Gran Canaria, 35017 Las Palmas de Gran Canaria, Spain; 2 Telematic Engineering Department, University of Las Palmas de Gran Canaria, 35017 Las Palmas de Gran Canaria, Spain; 3 Signal and Communications Department, University of Las Palmas de Gran Canaria, 35017 Las Palmas de Gran Canaria, Spain

**Keywords:** channel state information, Wi-Fi, human presence detection, classification learner

## Abstract

In recent times, we have been witnessing the development of multiple applications and deployment of services through the indoors location of people as it allows the development of services of interest in areas related mainly to security, guiding people, or offering services depending on their localization. On the other hand, at present, the deployment of Wi-Fi networks is so advanced that a network can be found almost anywhere. In addition, security systems are more demanded and are implemented in many buildings. Thus, in order to provide a non intrusive presence detection system, in this manuscript, the development of a methodology is proposed which is able to detect human presence through the channel state information (CSI) of wireless communication networks based on the 802.11n standard. One of the main contributions of this standard is multiple-input multiple-output (MIMO) with orthogonal frequency division multiplexing (OFDM). This makes it possible to obtain channel state information for each subcarrier. In order to implement this methodology, an analysis and feature extraction in time-domain of CSI is carried out, and it is validated using different classification models trained through a series of samples that were captured in two different environments. The experiments show that the methodology presented in this manuscript obtains an average accuracy above 90%.

## 1. Introduction

Traditional presence detection systems usually make use of magnetic opening sensors or special video surveillance devices, such as cameras or infrared motion detectors. In addition to the typical systems, which are composed of visible light cameras as the system developed in [1], different alternatives have been used, those that exploit passive Radio Frequency Identification (RFID) technology for this purpose such as the authors in [2] or infrared light, such as the joint use of visible light and infrared light images as explained in [3] or the system developed in [4] for infrared pedestrian detection.

Bluetooth low energy (BLE) technology is also a very common low-cost and widely used, and there have been multiple occupant detection studies using BLE systems such as [5], a system composed of BLE beacons installed inside the building, a mobile application installed on occupant’s mobile phones and a remote control server, yielding high classification accuracy for different numbers of installed beacons and occupant movement patterns. In [6], a scalable and less intrusive system that takes advantage of smartphone BLE technology to carry out occupant location at the zone level, requiring no application installed on the phone is carried out. In addition, in [7], a hybrid method fusing sliding-window filtering, trilateration, dead reckoning and the Kalman filtering method to improve the performance of the BLE indoor positioning is proposed.

In the building domain, there are multiple human presence detection systems that have been proposed to enable some real-world applications such as building emergency management. In addition, in [8], a novel IoT-based occupancy-driven plug load management system is proposed. In [9], a system is presented that leverages existing WiFi infrastructure in commercial buildings, along with smartphones with WiFi connectivity carried by building occupants, to provide precise occupancy-based climate control. Furthermore, in [10], an occupancy prediction is carried out based on a minimum sensing strategy by using a comprehensive set of sensor data to identify the most crucial features.

On the other hand, Wi-Fi-based solutions have also become popular due to their low cost and wide deployment. Although some of the mentioned passive presence detection technologies do not require special equipment and are low-cost and easy to implement, there are some existing challenges to resolve.

The possibility of developing presence detection systems based on Wi-Fi network is a viable solution without the use of any additional device, such as the system developed in [11]. In addition, in [12], a system to locate breathing in a home employing commercial Wi-Fi devices is implemented. This is partly due to the fact that no additional hardware is required and that, since the emergence of the IEEE 802.11n standard, additional channel information can be obtained.

On the other hand, CSI represents most detailed physical layer information compared to RSSI and BLE, providing channel information such as amplitude and phase on the subcarriers. CSI has been employed for person localization as can be seen in [13,14,15], for sign detection developed in [16], gesture detection in [17], human activity recognition as shown in [18,19], and person recognition such as WifiU [20] analyzes unique variations in the CSI on the Wi-Fi receiver, and even intrusion detection such as APID in [21]. CSI does not require any additional applications or use additional devices to detect human presence, unlike BLE deployments that usually require the installation of several BLE beacons in different locations to function properly, as the coverage area of BLE beacons is smaller than WiFi zones.

The most relevant contributions of this manuscript are as follows:

- CSI is used instead of RSSI because it provides more detailed physical layer information and can be used for location and tracking services with higher accuracy.
- Exploiting the information of the subcarriers amplitude provided by CSI and a feature extraction our approach is able to detect human presence in different environments.
- Only Wi-Fi networks are employed to detect human presence, and no additional hardware is needed.
- Classifiers evaluation is carried out, such as Support Vector Machine (SVM), decision trees and K-nearest neighbors (KNN) in order to detect which one can return a more accurate system.

This article is organized as follows: Section 2 summarizes the related work about presence detection systems employing CSI. Section 3 presents the CSI and its behavior. After that, Section 4 introduces the methodology to carry and the human presence detection system employed. Section 5 describes the scenarios and datasets that were built. Next, Section 6, the setup of the classifiers, an analysis of the different results and a comparison of the different classifiers is carried out. Finally, in Section 7, the conclusions and future work are presented.

## 2. Related Work

In recent years, a lot of research on indoor localization, fall detection and even gesture detection using CSI provided by a Wi-Fi network interface card (NIC) can be found in the literature. Starting by a fall detection system, in [22], the authors use CSI of the physical layer as an activity indicator. They can detect human falls without hardware modifications and without any handheld device, just by analyzing the CSI generated from an AP to a computer. The work starts by addressing the wireless radio propagation model in indoor environments while there are human activities, analyzing the radio propagation during falling and presenting a model when the user is at the Non Line Of Sight location.

The variation of the signal in the time-domain caused by human presence or movement and detection of the direction of intrusion are analyzed in [23]. This is based on the observation that the phase difference over two antennas shows fascinating characteristics in the presence of fall and other human activities. They propose a transition-based segmentation method that uses the variance of the phase difference over a pair of receiving antennas as a salient feature to segment on fall activities. Then, the characteristics of the CSI amplitude and phase information are extracted to distinguish between fall and fall-like activities.

In addition, in [24], the authors presented a real-time detection system without calibration based on Wi-Fi. They present a Fine-grained Indoor Motion Detection (FIMD) system that provides high accuracy, employing commercial 802.11n NICs to collect CSI samples by detection points fixed in the area of interest. After results evaluation from real world experiments, they demonstrate that the CSI-based motion detection by FIMD can improve detection accuracy.

In [25], authors established a scalable and non-intrusive solution for motion detection in smart home environments replacing existing Passive Infrared (PIR) sensors with Raspberry Pi 4 using CSI for accurate course movement detection. Their system aims to detect movement using variations in the correlation of sequential CSI frames. Measuring periods of increased variation that are identified as being caused by collisions on the subject’s body, and applying statistical approaches, they can effectively perform both movement and occupancy detection.

Authors in [26] proposed that the phase is more sensitive than the amplitude when there is an intruder. They focus on extracting the meaningful phase information, eliminating the significant random noise by employing linear transformation on the raw CSI. They propose a eigenvalue of covariance matrix of normalized CSI. Its characteristics are designed to be power-irrelevant but variation-dependent. Lastly, they adopt the dynamic time window algorithm on the sequence of CSI eigenvalues to detect intrusion behaviors.

In [27], a device free PAsive Detection of moving humans with dynamic Speed (PADS) was proposed. Exploiting both amplitude and phase provided by CSI. First, they obtain meaningful phase information by employing a linear transformation on the raw CSI. Next, they extract a new unified feature from the normalized amplitude and phase information, and then introduce the SVM algorithm to find a cut-off line of the feature values for different states for estimation. Finally, to improve the detection accuracy, they implement CSI across multiple antennas in MIMO.

In [28], the authors proposed a multi-room presence detection system in which only a transmitter is required, and the number of receivers depends on the number of rooms. With their proposed C-MuRP system, they obtained an accuracy of over 90%.

On the other hand, a Wi-Fi CSI based passive human intrusion direction detection system is developed in [29]. They propose an algorithm to monitor changes in amplitude and angle of arrival distributions of reflections and perform human intrusion detection.

The authors in [30] propose a process that goes through different steps in order to establish a presence detection classifier and the relationship between CSI fingerprints and locations, and classification to detect presence or regression to estimate locations.

As can be observed, there has been considerable progress in the advancement of approaches for human detection, using CSI to deal with the amplitude and phase information of subcarriers in OFDM systems or combining it with RSS information. In our system, only the amplitude given by the CSI is considered to analyze different scenarios and check when a human intrusion occurred by analyzing some parameters of the signal. In this study, a simple and accurate methodology is presented yielding an average accuracy over 90%, and employing time domain features such as the mean, standard deviation (STD), root mean square (RMS), and the number of changes that occurred in the signal.

## 3. Channel State Information

In all wireless communication, the signal propagates from the transmitter to the receiver. This signal is affected by the elements that are present in the environment causing reflections and diffractions due to the multi-path effect. The OFDM scheme employs orthogonal multiple subcarrier modulation. Each subcarrier carries information and suffers from multi-path effects such as delay, attenuation and phase shift. CSI is the information of these subcarriers and its representation in Channel Frequency Response (CFR) is given by the following equation:

(1) $$H\left(f;t\right)=\sum_{n}^{N}a_{n}\left(t\right)e^{-j2\pi f\tau_{n}\left(t\right)}$$

where *a(t)* is the amplitude attenuation factor, $\tau_{n}\left(t\right)$ is the propagation delay, and *f* is the carrier frequency [31].

Movements of objects or people as well as presence of people directly influence the amplitude and phase of each subcarrier. This feature can be exploited in applications such as people detection as well as indoor location.

The OFDM technique enables simple least squares channel estimation for CSI per subcarrier in an efficient way in the frequency domain. The MIMO system allows for more pairs of CSI observations with multiple transmit and receive antennas. In the frequency domain, the channel model can be expressed as:

(2) $$y_{k,l}=h_{k,l}*x_{k,l}+n_{k,l},$$

where $x_{k,l}$ and $y_{k,l}$ are the Tx and Rx signal at *k*-th subcarrier of *l*-th antenna pair, respectively. $h_{k,l}$ and $n_{k,l}$ are the channel response and additive white Gaussian noise. In the OFDM system, the CSI of the *k*-th subcarrier and the *l*-th antenna pair can be estimated as:

(3) $${\hat{h}}_{k,l}=\frac{y_{k,l}}{x_{k,l}}.$$

The CSI matrix can be expressed as:

(4) $$\hat{H}=\left[\begin{array}{cccc} {\hat{h}}_{1,1} & {\hat{h}}_{1,2} & \ldots & {\hat{h}}_{1,L}\\ {\hat{h}}_{2,1} & {\hat{h}}_{2,2} & \ldots & {\hat{h}}_{2,L}\\ \vdots & \vdots & \ddots & \vdots \\ {\hat{h}}_{K,1} & {\hat{h}}_{K,2} & \ldots & {\hat{h}}_{K,L} \end{array}\right]$$

where *K* is the number of subcarriers and *L* the antenna pair. Each element of the CSI matrix is represented as:

(5) $${\hat{h}}_{k,l}=\left|{\hat{h}}_{k,l}\right|e^{j\left(∠{\hat{h}}_{k,l}\right)}$$

## 4. Methodology

**Figure 1:**
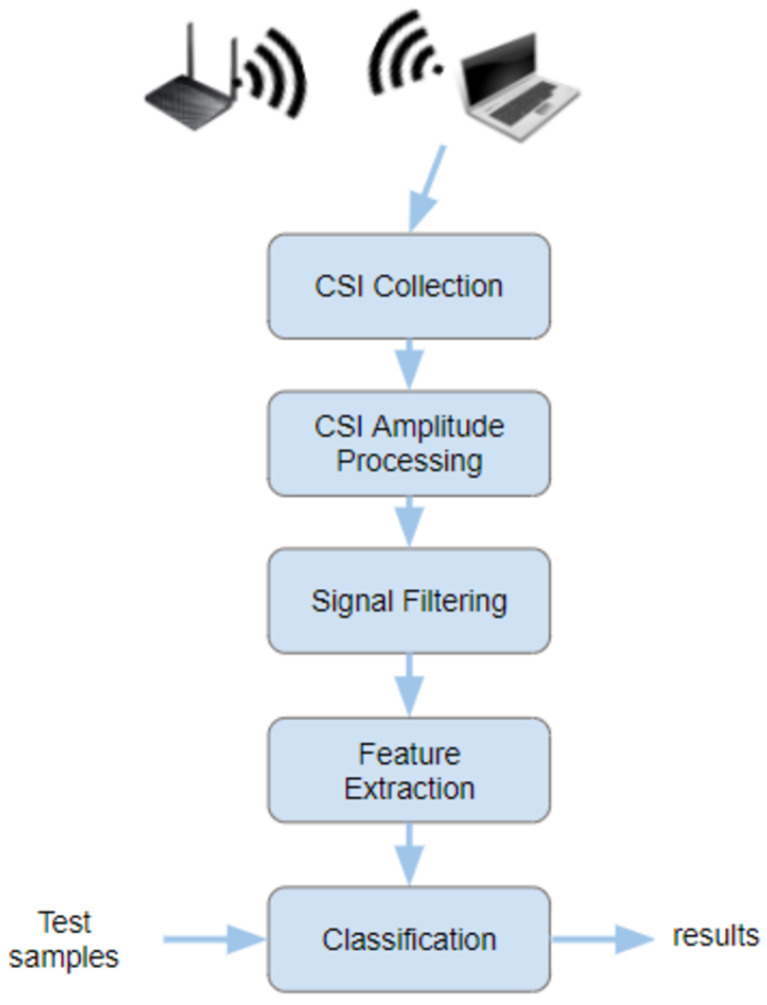
The proposed methodology with amplitude extraction.

The methodology followed in this research work is presented in Figure 1, which is developed in five stages: data collection, CSI amplitude processing, filtering, feature extraction and classification.

The process followed in this methodology can be briefly introduced as follows:

- First data are collected at every location previously defined from an IEEE 802.11 access point. CSI information specifies the amplitude and phase of the signal path between a single transmitter–receiver antenna pair.
- In the next step, CSI amplitude processing, where CSI is processed to extract subcarriers amplitude for the transmission. After that, these signals are filtered to achieve better results with a low-pass filter implemented with a MATLAB function.
- Next, the dataset is built with these vectors feature extraction.
- In addition, finally, they are used as an input of the machine learning algorithm to create the different models.

Each stage is described in the following sub-sections.

### 4.1. Data Collection

In order to analyze the influence of a human presence in the transmission, the samples are captured in a known scenario. First, the system was configured so it worked at a 2.4 GHz WiFi band which was the supported by the access point. In that scenario, the position for the AP and the computer that capture the CSI is defined. In addition, after setting the position of the computer and the AP, the process of collecting samples without any human interfering is carried out. Once enough samples had been taken, additional samples are collected with human presence.

The process of capturing the human presence was carried out with different delays, 10 s, 20 s and about 30 s after the start.

### 4.2. CSI Amplitude Processing

**Figure 2:**
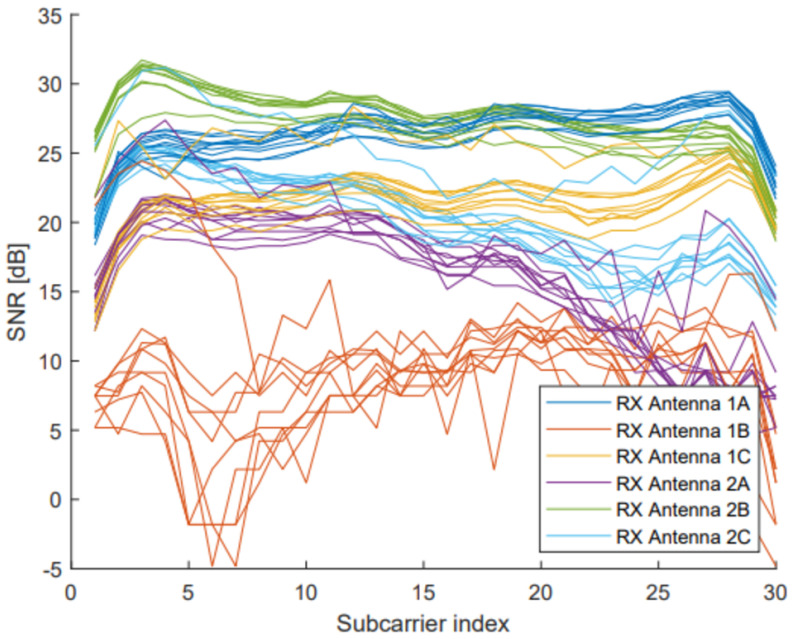
Amplitude representation in a 2 × 3 communication.

In MIMO communication, not all receiver antennas are always used. In addition, the Tx–Rx antenna pair with the highest subcarrier amplitude has the highest stability. Figure 2 shows the amplitude of a set of samples, for each Tx–Rx antenna pair in a 2 × 3 communication.

As can be seen, each pair returns different values, and therefore it was necessary to make a selection of which pair would be used in the different tests. To select the best pair, the average of each pair is computed as:

(6) $$\left|CSI\right|=\frac{1}{S}\sum_{i=1}^{S}\sqrt{{\left|H_{i}\right|}^{2}+∠H_{i}^{2}}$$

where *S* is the number of subcarrier, and $∠H_{i}$ and $\left|H_{i}\right|$ represent the phase and amplitude information of the *i*-th subcarrier, respectively. This information is provided as a complex number, and therefore, $\left|CSI\right|$ represents the averaged magnitudes of *S* complex numbers. Once the $\left|CSI\right|$ value of each pair is estimated, the pair with the highest amplitude value is selected. This pair is selected because it is the one with the highest stability, which allows a parameter extraction to be clearer when human presence is detected.

### 4.3. Signal Filtering

After the process of selection in which the Tx–Rx antenna pair is selected, a low pass filter is applied to reduce the noise added to the signal; this is also implemented with the MATLAB function *lowpass(x, wpass)*.

Where *x* is the CSI amplitude matrix and *wpass* is the normalized pass band frequency. This function filters the input signal *x* using a *lowpass* filter with normalized passband frequency *wpass* in units of πrad/sample. This function uses a minimum-order filter with a stopband attenuation of 60 dB and compensates for the delay introduced by the filter. This filter is required in order to attenuate the effect of possible interferences in our signal. In our methodology, we use a 0.5 value as a *wpass* parameter because it provided the best results at the classification phase.

### 4.4. Features Extraction

To carry out the classification, an evaluation of some features in time domain was implemented, such as the mean value, the number of relevant changes according to a threshold, the standard deviation, the quadratic mean, and the kurtosis. The combination of the first four indicated parameters obtained the best performance results in the classification phase. These parameters have been selected because they are fast to obtain and do not require much time to process. With these values, the following parameters are obtained:

- Max Mean: gives the maximum value of the mean in the sample of amplitude information;
- Max RMS: gives the maximum value of the root mean square in the sample;
- NumberOfChanges: show the number of abrupt changes in the signal;
- Max STD: gives the maximum value of the standard deviation in the signal.

**Figure 3:**
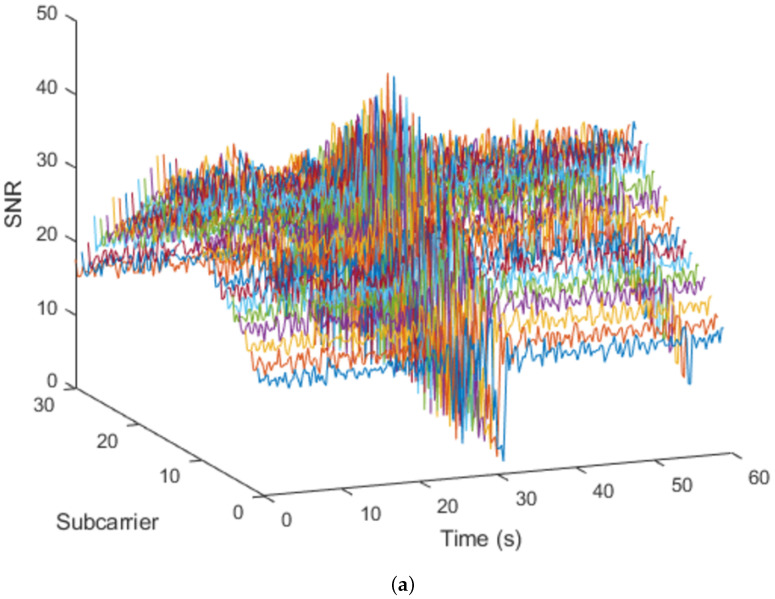

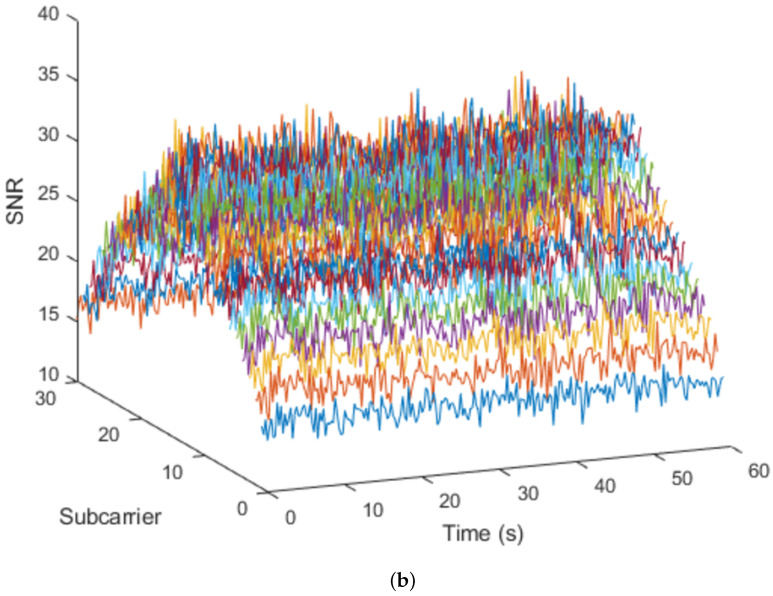
CSI amplitude representation: (**a**) with human presence; (**b**) without human presence.

Table 1 shows the parameters when there is a human presence besides those in which there is not. These parameters are related with the samples shown in Figure 3 that displays the difference between a signal that have been affected by an intrusion, on the left, and a signal without interference, on the right.

**Figure 4:**
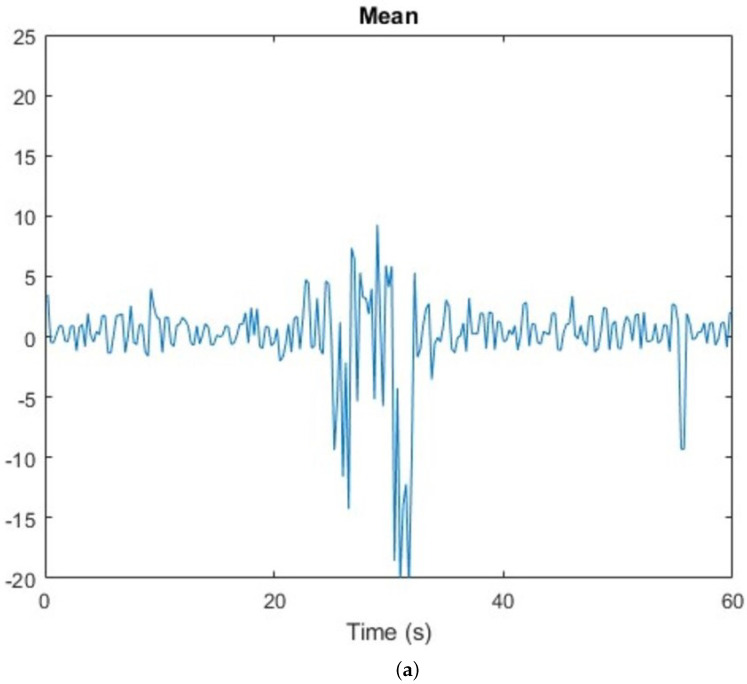

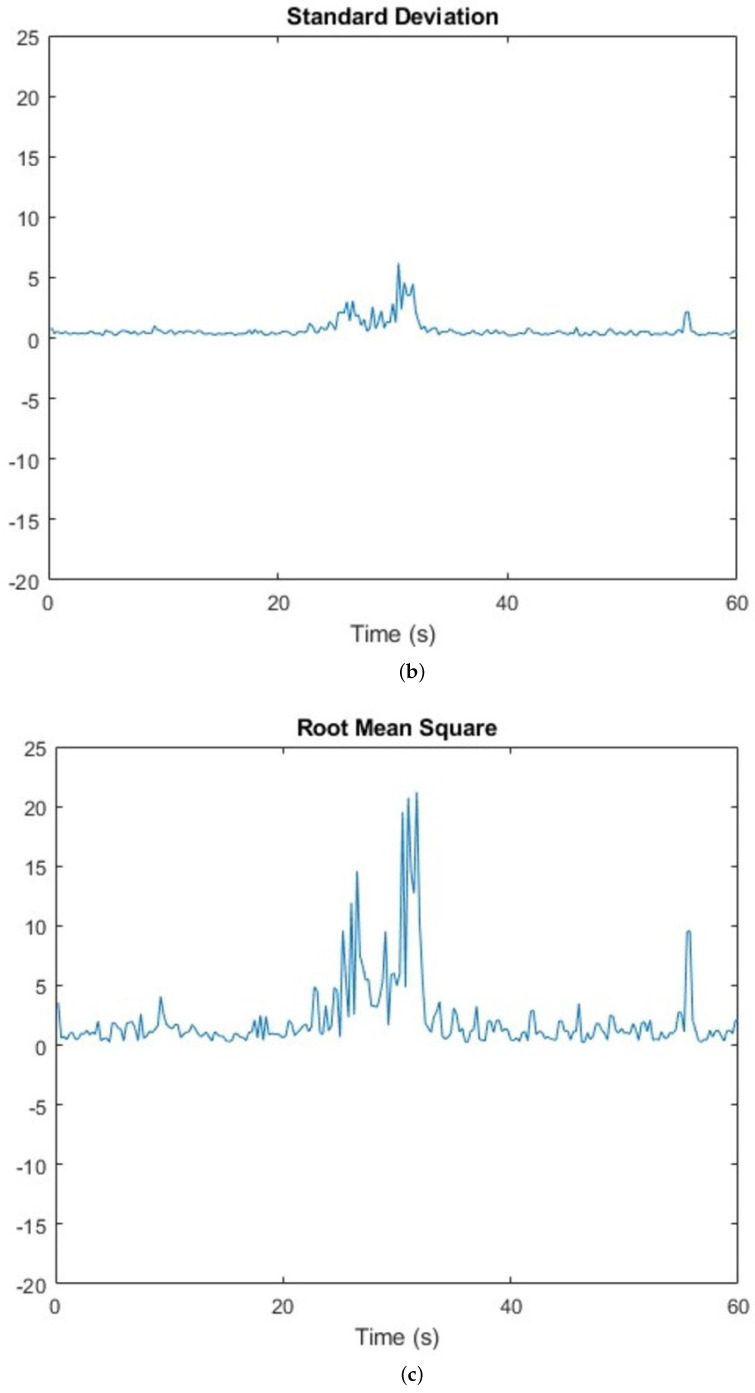

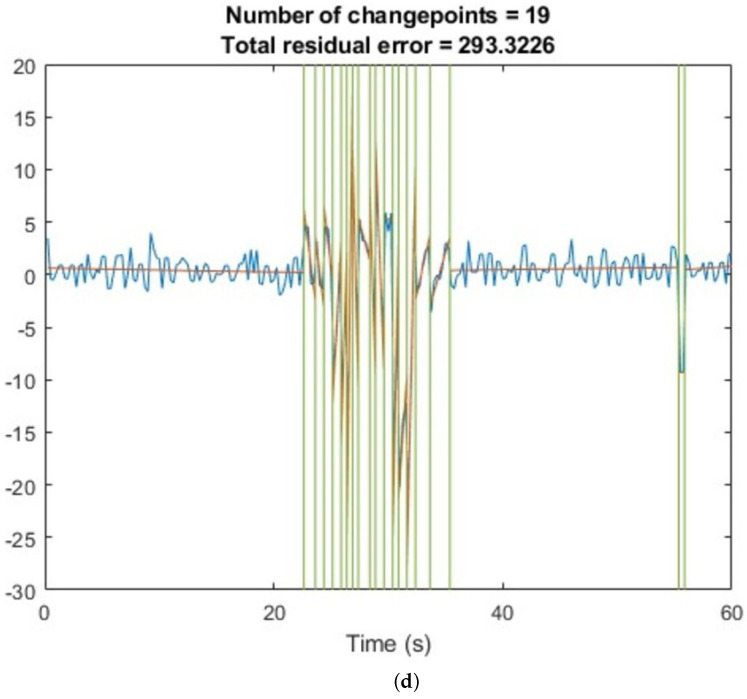
Parameter setting results for a human presence detection: (**a**) mean representation; (**b**) STD representation; (**c**) RMS representation; (**d**) number of changes in the signal.

In addition, a human presence detection is shown in Figure 4. First, there is no human presence in the space being captured, then human access in the scenario about 25 s from starting data collection, and finally there is no human presence again.

As can be observed from Table 1 and Figure 4, the parameters suffer a high variation when a human interferes in a scenario.

### 4.5. Classification

Our methodology attempts to detect a human presence, and to achieve this, two different categories were created which are “Presence” that refers to when is a human presence and “No-Presence” that means that there was no human in the environment. In order to validate this data, different machine learning algorithms were used such as K-Nearest Neighbors (KNN), SVM, Decision trees, Linear discrimination, Quadratic discrimination, Naive Bayes, Neuronal Networks and Ensemble classifiers. There are different types of some of these classifiers that were studied such as Linear SVM or Quadratic SVM. Furthermore, in the case of the KNN classifiers, different models with a different number of neighbors were trained. The amount of neighbors selected was 1, 10 and 100. In addition, there were models with different distance weight formulas and distance metric. A total of 31 classification models have been studied, but only the classifiers with the best accuracy will be analyzed.

## 5. Datasets

Three datasets have been employed in order to validate this methodology. These datasets contain CSI measurements collected from a single IEEE 802.11n access point.

An Acer Travelmate 6293 laptop with Intel WiFi Link 5300 802.11n wireless network card was used to gather CSI measurements from the access point, which was configured using the Linux 802.11n CSI tool developed by [32]. This laptop has three internal antennas for wireless communications. The CSI tool provides CSI values in a format that reports the channel matrices for 30 subcarrier groups for every two subcarriers at 20 MHz. Each matrix entry is a complex number specifying amplitude and phase of the signal path between a Tx–Rx antenna pair.

On the other hand, an ASUS RT-N12E Wireless-N300 access point with two antennas was used. Therefore, since in both scenarios a 2 × 3 MIMO communication can be achieved, 90 raw CSI measurements (1 × 3 MIMO transmission) can be collected for each packet reception. This means that 30 raw CSI measures are collected from each receiver antenna.

### 5.1. Scenario 1

**Figure 5:**
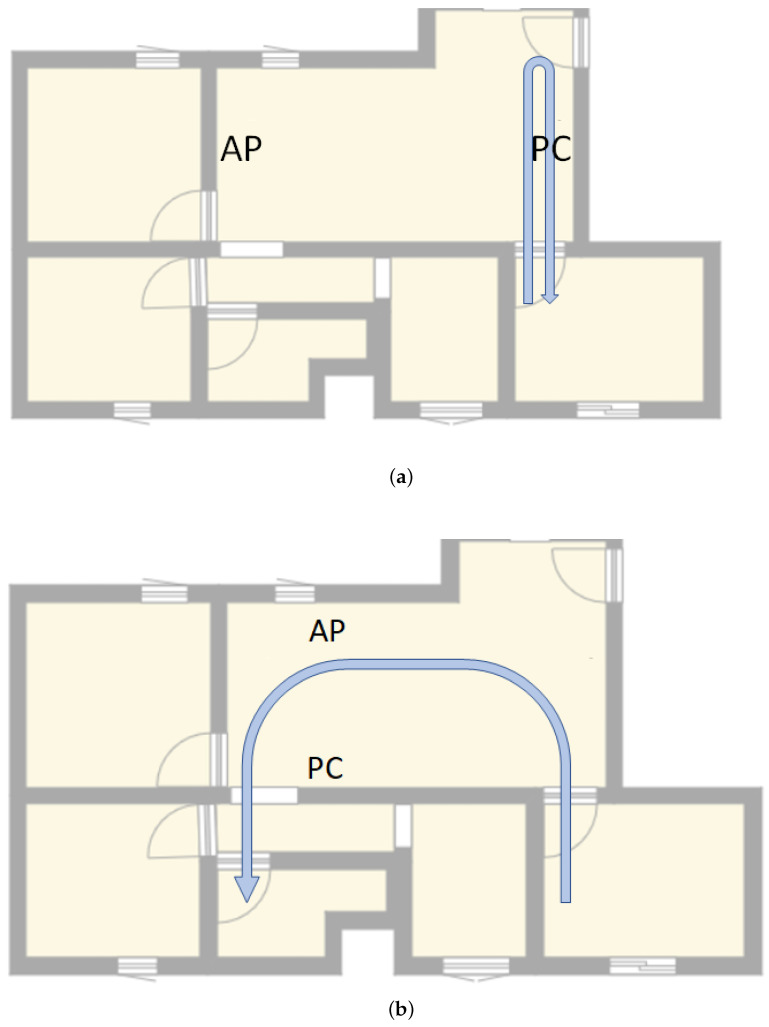
Paths taken in scenario 1: (**a**) returning to the initial position; (**b**) ending the path in a different room.

In this scenario, samples were taken placing the devices in two different places of the room and interfering with two different routes, as shown in Figure 5. In this case, a total of 264 samples were taken, 139 were taken without any presence, while the remaining 125 samples had a human presence interfering with the transmission signal for a period of time.

### 5.2. Scenario 2

**Figure 6:**
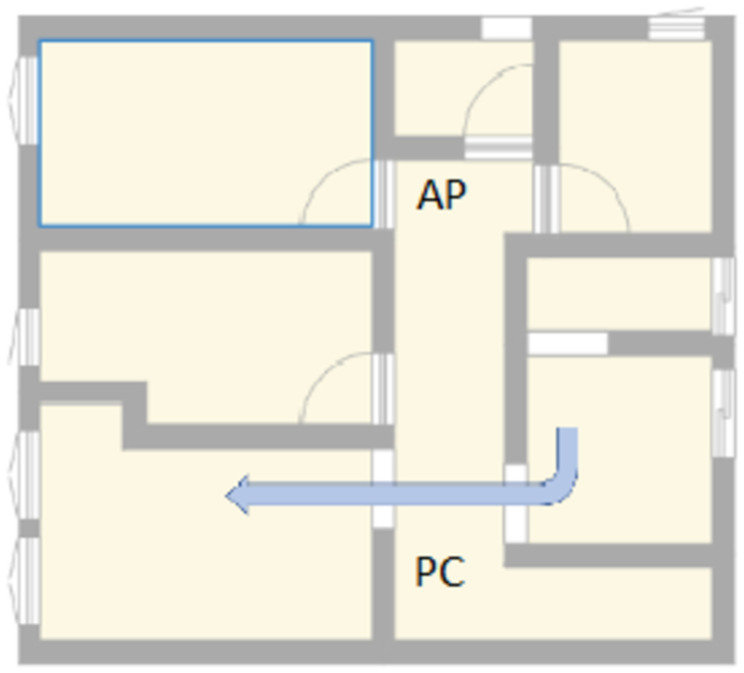
Scenario 2.

This dataset was taken in a placed named as scenario 2, with 115 samples taken, 65 without any presence and the remaining samples with a human presence walking through the access point and the computer. In Figure 6, the path traveled by the person and the position of the access point and the computer are shown.

### 5.3. Both Scenarios

This dataset was created after all samples were taken and contains all samples taken in scenario 1 and scenario 2. In total, it contains 379 samples, of which 204 are samples with no human presence and 175 are samples that were interfered with by a human for a period of time.

## 6. Results and Discussion

In order to make the classification the easier and fastest, MATLAB classification learner app was employed because it can perform automated training to search for the best classification model type, including decision trees, discriminant analysis, support vector machines, logistic regression, nearest neighbors, naive Bayes, kernel approximation, ensemble, and neural network classification.

Since a different configuration for Tx–Rx antennas can be configured and, depending on which pair is selected, the amplitude values of the signal can differ. To select which pair was going to be used for the classification, all possible pairs were tested, and the results both graphically and numerically were evaluated. The pair Tx-2 and Rx-2 was selected after a study comparing the results for the six possible antenna pairs because it was the one that returned the most easily distinguishable values when there is human presence and when there is not. After this evaluation, all the tests and classifications were carried out with this pair.

In order to analyze the samples in each of the datasets described in Section 5, simulations were carried out implementing the 10-fold cross-validation technique. This means that in each experiment the data set is divided into 10 partitions, and 10 rounds are performed in which 9 partitions are used for training and 1 for testing. In addition, the results that are going to be analyzed are for the 3 best classifiers over the 31 that were trained.

### 6.1. Scenario 1

In Table 2, the results for the best 3 classifiers are shown.

As shown by the results, we can see how, depending on the percentage of samples used for the training of the model, the accuracy is higher when more samples are used. Moreover, in this case, we can conclude that the most accurate classifier is the linear SVM model with an average accuracy of 92.99% with an STD of 0.58.

### 6.2. Scenario 2

As in the previous scenario, in Table 3, the results for the best 3 classifiers are shown.

Analyzing the results for this scenario, as in the other scenario, the highest accuracy is given by the linear SVM model with an average accuracy of 93.58% and a STD of 0.57 when it is trained with 75% of the samples. In this scenario, the other classifiers obtained values that can outperform the linear SVM when trained with fewer samples, but as the number of samples used for training increases, their accuracy decreases. The only one that increased its accuracy when more samples are used is the linear SVM.

### 6.3. Both Scenarios

When both scenarios were analyzed simultaneously, the best classifiers were the same that are in scenario 2. However, in this case, the Quadratic discrimination model was not the best. The accuracy for this classifiers is shown in Table 4.

In this case, where samples from both scenarios are used for training, as in the previous datasets that were analyzed, the one with the highest accuracy is the linear SVM model, with an average accuracy of 93.24% and a STD of 0.31.

### 6.4. Performance Evaluation

Analyzing the whole datasets and its results for each of them, we can conclude which classifier is the best for each one. Reviewing the results of each scenarios, it can be concluded that the best average accuracy is given by the linear SVM; in Table 5, we can check the results of this classifier for each dataset.

As can be seen by the results obtained in all the different scenarios for this classifier model, when more samples are used for training, better results are obtained. In addition, when these results are compared with the other two models with the highest accuracy, this one is the only one that obtained an increasing accuracy when more samples are used for training, which is the expected result, since, when more samples are used, it should be clearer which samples contain a human presence. The other two classifier models sometimes show higher accuracy when trained with fewer samples, and may decrease in accuracy when given more samples to train.

## 7. Conclusions and Future Work

In this manuscript, a methodology using the CSI amplitude provided in Wi-Fi networks to infer the human presence is described. This methodology is based on features in time-domain in order to carry out the human presence detection. The evaluation of the proposed methodology indicates that deployed Wi-Fi networks can be used for intrusion event, leading to satisfactory accuracy. The proposal was verified and validated in two different real-world scenarios.

As shown in Section 6, we conclude that the linear SVM classifier was the most suitable in this case and scenario since it has an accuracy rate over 92% in all the scenarios and tests. In addition, we can conclude that, with this parameter setting and the classification learner app, it is possible to determine when a human interference is occurring.

One of the limitations of the system is that it is only able to detect one intrusion at a time, and it is not able to distinguish whether the intrusion has been caused by one or several people. In addition, if a previous and analyzed environment is modified, new samples are needed to retrain the classification models, since the values may vary from those previously obtained. In our ongoing work, one of the improvements that can be implemented is a model which is able to distinguish whether over time there is one or more intrusions by capturing the samples because, if an interference is performed in different time periods, it can be easily distinguishable. In addition, further parameterization of the CSI signal, including its phase, could be analyzed, which could address some of the limitations of the system and improve the accuracy rate of the classifiers. Furthermore, a study of several deep learning techniques employing different activation functions could be performed to evaluate the results of different classifiers over those studied aforementioned.

## Figures and Tables

**Table 1:** Example of parameter results when there is human presence, and without it.

Max Mean	Max RMS	Number of Changes	Max STD	SIGNAL
9.2722	21.1972	19	6.1864	‘Presence’
3.4004	3.5627	1	1.0912	‘No-Presence’

**Table 2:** Scenario 1 best classifiers.

Models	25%	50%	75%
	Mean ± STD	Mean ± STD	Mean ± STD
Linear SVM	89.71 ± 3.76	92.4 ± 0	92.99 ± 0.58
Quadratic Discrimination	90.9 ± 0.95	90.73 ± 0.6	92.7 ± 0.25
Quadratic SVM	88.49 ± 1.2	90.29 ± 1.2	91.4 ± 0.84

**Table 3:** Scenario 2 best results.

Models	25%	50%	75%
	Mean ± STD	Mean ± STD	Mean ± STD
Linear SVM	91.67 ± 2.76	91.74 ± 0.69	93.58 ± 0.57
Quadratic Discrimination	95.18 ± 1.71	92.41 ± 0.83	91.05 ± 0.86
Quadratic SVM	93.08 ± 3.08	88.95 ± 1.41	91.73 ± 0.86

**Table 4:** Best results obtained with all the samples.

Models	25%	50%	75%
	Mean ± STD	Mean ± STD	Mean ± STD
Linear SVM	90.35 ± 1.47	93.4 ± 0.25	93.24 ± 0.31
Quadratic Discrimination	90.37 ± 0.60	93.79 ± 0.37	92.36 ± 0.22
Quadratic SVM	84.2 ± 4.15	93.14 ± 0.54	92.92 ± 0.45

**Table 5:** Linear SVM classifier results.

Models	25%	50%	75%
	Mean ± STD	Mean ± STD	Mean ± STD
Scenario 1	89.71 ± 3.76	92.4 ± 0	92.99 ± 0.58
Scenario 2	91.67 ± 2.76	91.74 ± 0.69	93.58 ± 0.57
Both Scenarios	90.35 ± 1.47	93.4 ± 0.25	93.24 ± 0.31

## Data Availability

Not applicable.

## Abbreviations

The following abbreviations are used in this manuscript:

BLE	Bluetooth Low Energy
CFR	Channel Frequency Response
CSI	Channel State Information
FMID	Fine-Grained Indoor Motion Detection
IEEE	Institute of Electrical and Electronics Engineers
KNN	K-Nearest Neighbors
MIMO	Multiple Input Multiple Output
NIC	Network Interface Card
OFDM	Orthogonal Frequency Division Multiplexing
PIR	Passive Infrared
RFID	Radio Frequency Identification
RMS	Root Mean Square
RSS	Received signal strength
RSSI	Received Signal Strength Indicator
Rx	Receiver
STD	Standard Deviation
SVM	Support-Vector Machines
Tx	Transmitter
